# TNFα Modulates Cardiac Conduction by Altering Electrical Coupling between Myocytes

**DOI:** 10.3389/fphys.2017.00334

**Published:** 2017-05-23

**Authors:** Sharon A. George, Patrick J. Calhoun, Robert G. Gourdie, James W. Smyth, Steven Poelzing

**Affiliations:** ^1^Department of Biomedical Engineering and Sciences, Virginia Polytechnic Institute and State UniversityBlacksburg, VA, United States; ^2^Department of Biological Sciences, Virginia Polytechnic Institute and State UniversityBlacksburg, VA, United States; ^3^Center for Heart and Regenerative Medicine, Virginia Tech Carilion Research InstituteRoanoke, VA, United States

**Keywords:** TNFα, conduction, calcium, ephaptic coupling, connexin43

## Abstract

**Background:** Tumor Necrosis Factor α (TNFα) upregulation during acute inflammatory response has been associated with numerous cardiac effects including modulating Connexin43 and vascular permeability. This may in turn alter cardiac gap junctional (GJ) coupling and extracellular volume (ephaptic coupling) respectively. We hypothesized that acute exposure to pathophysiological TNFα levels can modulate conduction velocity (CV) in the heart by altering electrical coupling: GJ and ephaptic.

**Methods and Results:** Hearts were optically mapped to determine CV from control, TNFα and TNFα + high calcium (2.5 vs. 1.25 mM) treated guinea pig hearts over 90 mins. Transmission electron microscopy was performed to measure changes in intercellular separation in the gap junction-adjacent extracellular nanodomain—perinexus (W_P_). Cx43 expression and phosphorylation were determined by Western blotting and Cx43 distribution by confocal immunofluorescence. At 90 mins, longitudinal and transverse CV (CV_L_ and CV_T_, respectively) increased with control Tyrode perfusion but TNFα slowed CV_T_ alone relative to control and anisotropy of conduction increased, but not significantly. TNFα increased W_P_ relative to control at 90 mins, without significantly changing GJ coupling. Increasing extracellular calcium after 30 mins of just TNFα exposure increased CV_T_ within 15 mins. TNFα + high calcium also restored CV_T_ at 90 mins and reduced W_P_ to control values. Interestingly, TNFα + high calcium also improved GJ coupling at 90 mins, which along with reduced W_P_ may have contributed to increasing CV.

**Conclusions:** Elevating extracellular calcium during acute TNFα exposure reduces perinexal expansion, increases ephaptic, and GJ coupling, improves CV and may be a novel method for preventing inflammation induced CV slowing.

## Introduction

Myocardial inflammation is associated with many cardiac diseases (Marchant et al., 2012; De Jesus et al., 2015) and modulates several determinants of cardiac function, both mechanical and electrical. Mechanically, myocardial inflammation can cause cardiac dysfunction and reduced ejection fraction (Lurz et al., 2012; Banka et al., 2015). Myocardial inflammation can also alter electrical impulse propagation by modulating gap junctional coupling (GJC) (Zhu et al., 2000; Xu H. F. et al., 2012), ionic currents (Tang et al., 2007; De Jesus et al., 2015), and tissue hydration state (Logstrup et al., 2015).

Inflammation is a complex process associated with the modulation of several physiologic factors, including the up- and downregulation of many cytokines, which are cell signaling molecules (Zhang and An, 2007). Cytokines modulate numerous cellular processes, some pro-inflammatory and others anti-inflammatory. Tumor Necrosis Factor α (TNFα) is a pro-inflammatory cytokine whose upregulation is a key marker of the acute inflammatory phase in several pathophysiologic states including ischemia, myocarditis, and cardiomyopathies (Matsumori et al., 1994; Intiso et al., 2004). TNFα upregulation modulates the expression of other cytokines and has a cascading effect on the inflammatory process. The effects of TNFα on various cellular functions have been extensively studied in cardiac and other tissue types. For example, some studies (Celes et al., 2007; Kimura and Nishida, 2010) demonstrated that exposure to TNFα reduces Connexin43 (Cx43) functional expression, the principle gap junctional protein in cardiac ventricles, while others reported no change (Sawaya et al., 2007). Other studies determined that TNFα can modulate Cx43 phosphorylation states in anterior pituitary cells (Meilleur et al., 2007). Studies have also demonstrated a temporal change in the regulation of Cx43 expression by TNFα where an increase in Cx43 mRNA and protein expression was reported at 6 h of TNFα exposure and a decrease at longer durations up to 48 h (Liu et al., 2012). However, the electrophysiologic effects of acute TNFα exposure in cardiac tissue are not fully understood.

In addition to its effect on GJ coupling, TNFα can also modulate vascular permeability which can alter tissue hydration state (Hansen et al., 1994). However, it is not known how this translates to the level of intercellular separation at nanodomains within the intercalated disc, such as the gap junction adjacent perinexus (Rhett et al., 2011). Additionally, TNFα has also been reported to reduce the expression of structural proteins along the intercalated disc (ID) and cause ID dehiscence (Celes et al., 2007). Both factors could cause perinexal widening which is associated with CV slowing possibly due to weaker ephaptic coupling (EpC) between myocytes (George et al., 2015, 2016; Veeraraghavan et al., 2015). Therefore, TNFα alone can modulate various determinants of CV similar to previously reported models of myocardial inflammation. In this study, we use pathophysiologic TNFα exposure as a model for myocardial inflammation and focus on the acute effects of TNFα on ventricular conduction. We hypothesized, that TNFα modulates CV by reducing electrical coupling in the heart—both EpC and GJC.

Here, we determined the ventricular conduction velocity response to TNFα exposure (100 pg/ml) over 90 mins. Our results suggest that CV slows with TNFα exposure relative to control. CV slowing is associated with reduced EpC but no significant modulation of GJC. Elevating extracellular calcium ion concentration ([Ca^2+^]_o_) improved both forms of electrical coupling in the presence of TNFα, which could have contributed to restoring CV to control values.

## Methods

All experimental protocols have been approved by the Institutional Animal Care and Use Committee at Virginia Polytechnic Institute and State University and are in accordance with the NIH Guide for Care and Usage of Laboratory Animals.

### Landgendorff preparation

Adult male Hartley Guinea Pigs (1,000–1,300 g) were anesthetized by exposure to isoflurane and hearts were excised following thoracotomy as previously described (Veeraraghavan et al., 2015). The heart was then attached to a Langendorff perfusion system and perfused with a solution containing, in mM, 1.25 CaCl2, 140 NaCl, 5.5 NaOH, 4.5 KCl, 5.5 Dextrose, 0.7 MgCl2, 9.9 HEPES, pH 7.4 at 37°C. The atria were removed and the heart was suspended in a bath containing the same perfusate at 37°C. Pressure was maintained at ~50 mmHg.

### Optical mapping

After a 30 min stabilization period, hearts were perfused with 7.5 μM Di-4-ANEPPS for ~10 mins after which excess dye was washed out, which is *t* = 0 mins for all experiments. The electromechanical uncoupler, 2,3-butanedionemonoxime was added to the perfusate to reduce motion. A silver pacing wire was placed on the anterior ventricular surface of the heart in the center of the mapping field and a reference wire was introduced in the back of the bath. Hearts were stimulated at 1 V for 1 ms stimuli at a BCL of 300 ms. The dye was then excited by light at 510 nm and the emitted light was filtered by a 610 nm filter and captured by a Micam Ultima L-type CMOS camera as previously described (George et al., 2015; Entz et al., 2016).

Optical data were analyzed to measure CV—both longitudinal (CV_L_) and transverse (CV_T_), anisotropic ratio (AR = CV_L_/CV_T_), action potential duration (APD), and rise time (RT). Briefly, activation times were assigned at the maximum rate of rise of the action potential and were fitted to a parabolic surface to determine CV vectors. APD was defined as the time interval between activation time and 90% repolarization. RT was calculated as the time interval between 20 and 80% of the upstroke of the action potential.

Hearts were subjected to one of three interventions over 90 mins and optical recordings were obtained at 15 min intervals (*n* = 6 hearts for each of three intervention). In the first group (control) hearts were continuously perfused with control Tyrode for the entire 90 mins. In the second group (TNFα) hearts were perfused with control Tyrode + TNFα at 100 pg/ml for the 90 min duration. Finally, in the third group (TNFα + high calcium) hearts were perfused with control Tyrode + TNFα for the first 30 mins, followed by calcium elevation to 2.5 mM still in the presence of TNFα from *t* = 31 to 90 mins.

### Electrocardiography

Volume conducted ECGs were recorded by silver chloride electrodes placed in the bath. The signals were recorded using the PowerLab 4/35 data acquisition system and LabChart Pro software. Signals were sampled at 1,000 Hz and filtered (0.1 and 50 Hz low and high cut off frequencies) to remove noise. Paced QRS duration and QT intervals were measured every 15 mins.

### Transmission electron microscopy

Anterior epicardial tissue from the left ventricle (*n* = 3 hearts × 3 intervention × 15 images) was collected from hearts at *t* = 0 mins and after 90 mins during the three interventions—control, TNFα, and TNFα + high calcium, sliced into 1 mm^3^ sections, fixed in 2.5% glutaraldehyde overnight at 4°C and then washed and stored in PBS also at 4°C. Samples were then processed for TEM as previously described (George et al., 2015) and imaged using a JEM JEOL1400 Electron Microscope at X150,000 magnification. Fifteen images were acquired per sample, which were then analyzed using ImageJ to measure perinexal width. The average of six intermembrane distances between 30 and 105 nm away from the edge of the GJ plaque, 15 nm apart, is reported as W_*P*_. Data are reported at mean ± standard error.

### Western blotting

Samples (*n* = 3 hearts × 3 conditions × 3 runs) were snap frozen at *t* = 0 or after 90 mins of control, TNFα or TNFα + high calcium treatment and immunoblotting was performed as previously described (Smyth et al., 2010) to determine Cx43 and pCx43—Ser368 expression. Briefly, samples were homogenized in RIPA lysis buffer (50 mM Tris pH 7.4, 150 mM NaCl, 1 mM EDTA, 1% Triton X-100, 1% sodium deoxycholate, 2 mM NaF, 200 μM Na_3_VO_4_) supplemented with HALT protease and phosphatase inhibitors (ThermoFisher Scientific) and electrophoresis was performed to separate proteins which were then transferred onto a PVDF membrane. This was then blocked with 5% BSA for 1 h at room temperature, followed by incubation with pCx43-Ser368 primary antibody (1:1,000, #3511, Cell Signaling Technologies) overnight at 4°C and, after washing, secondary antibody (1:5,000, Goat Anti-Rabbit HRP, abcam) for 1 h at room temperature. Protein expression was then quantified by ECL assay using a BioRad Chemidoc MP system. The membranes were then stripped with ReBlot Plus Strong (EMD Millipore) as per manufacturer's instructions and blocked with 5% milk for 1 h at room temperature. Membranes were then incubated with primary anitbodies against Cx43 (1:3,000, C2619 rabbit, Sigma Aldrich) and GAPDH (1:3,000, T6199 mouse, Sigma Aldrich) overnight at 4°C, followed by the corresponding secondary anitbodies (both 1:1,000, goat anti-mouse AlexaFluor555 and goat anti-rabbit AlexaFluor647) for 1 h at room temperature. Finally, total Cx43 and GAPDH protein expression was quantified using the BioRad Chemidoc MP system. Total Cx43 was normalized to GAPDH and pCx43 was normalized to total Cx43.

### Confocal immunofluorescence

Ventricular sections from control (*n* = 3), TNFα (*n* = 6), and TNFα + high calcium (*n* = 3) hearts at *t* = 0 mins and after 90 mins were snap frozen in OCT. Samples were sectioned at 5 μm thickness onto glass slides and fixed with 2% paraformaldehyde for 5 mins on a rotator. Slides were then washed and samples were blocked with a solution containing 1% BSA and 1% Triton X-100 in PBS for 1 h at room temperature. Samples were then incubated with primary antibody against Cx43 (1:4,000, C2619 rabbit, Sigma Aldrich) and N-Cadherin (1:100, 610920, mouse, BD Biosciences) overnight at 4°C. Slides were then washed and samples were incubated with the corresponding secondary antibodies (1:4,000, Goat Anti-Rabbit AlexaFluor 488 and Goat Anti-Mouse AlexaFluor 633) for 2 h at room temperature. Prolong Gold Antifade (Life Technologies) was then applied to the slides and slide covers were applied. Slides were cured for ~48 h. Cx43 and N-Cadherin distribution were imaged using a Leica TCS SP8 laser scanning confocal microscope using a X63 oil immersion lens. Images acquired (3 per heart) were then analyzed similar to previously described methods. (Smyth et al., 2014; Yan et al., 2014) Briefly, images were converted to a binary format after thresholding, and the percent of Cx43 colocalized with N-Cadherin and normalized to total Cx43 was quantified to estimate Cx43 localization at the intercalated disc.

### Statistical analysis

Single factor or two way ANOVA tests were performed to detect significant differences in the data and Student's *t*-test was applied as a *post-hoc* analysis. Bonferroni correction was applied as necessary with multiple comparisons. All data are reported as mean ± standard deviation unless stated otherwise. *p* < 0.05 was reported as significant.

## Results

### Conduction velocity-control vs. TNFα

**Figure 1:**
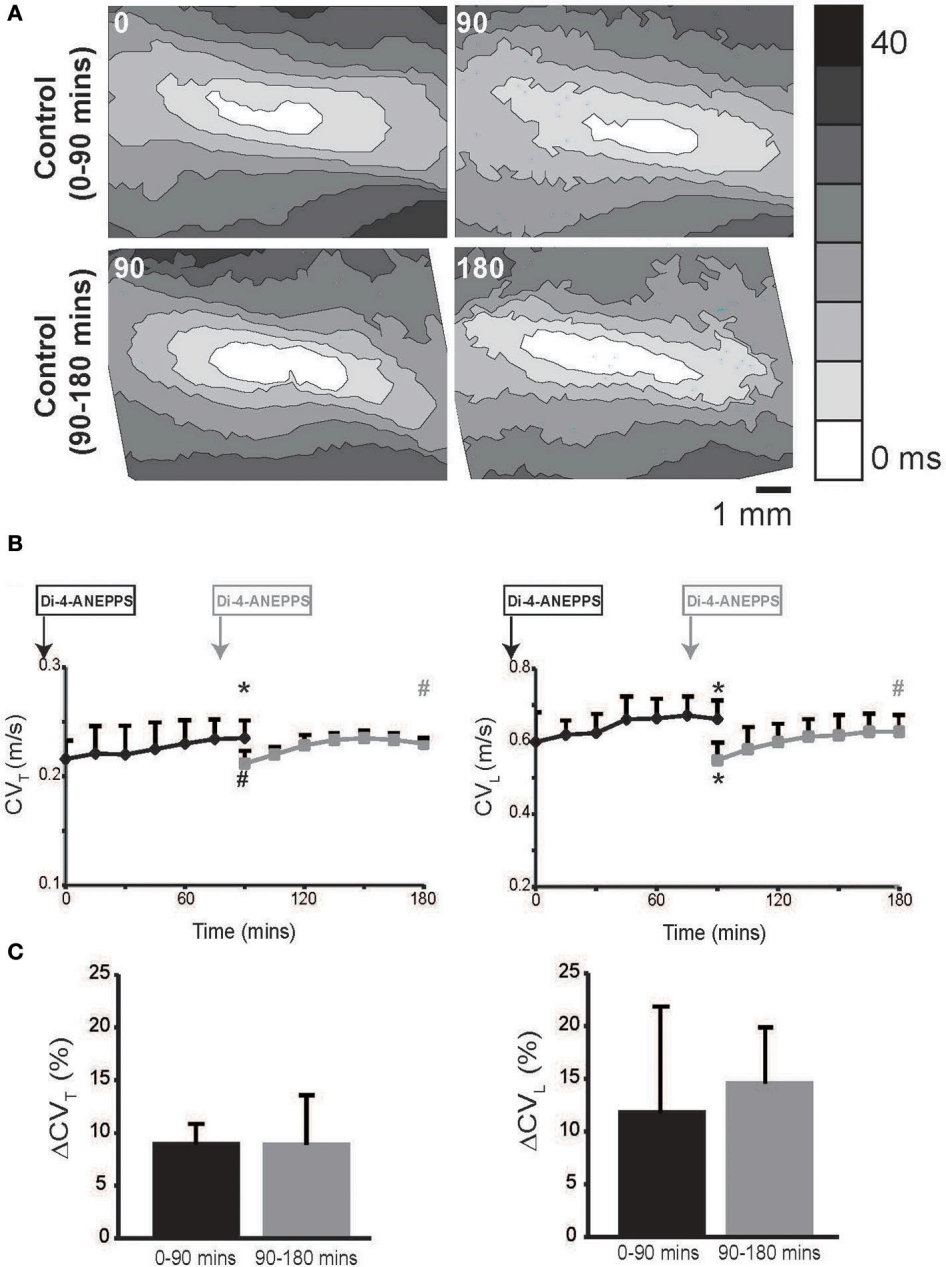
**Conduction velocity quantified by optical mapping increases with time. (A)** Representative isochrone maps from control Tyrode's solution perfused hearts at 0, 90 and 90, 180 mins. **(B)** Summary CV_L_ and CV_T_ measured up to 180 mins. Black curves indicate experiments where dye was perfused before *t* = 0 mins and gray curves indicates experiments where dye was perfused at 75 mins. **(C)** Percent increase in CV_T_ and CV_L_ over 90 min durations in the two sets of experiments. Black and gray ^*^Indicates *p* < 0.05 relative to *t* = 0 or 90 mins respectively by paired comparison. Black # indicates *p* < 0.1 comparing between black and gray data point at 90 mins. Gray # indicates *p* < 0.1 comparing gray data point 90 and 180 mins.

Hearts were optically mapped during perfusion of control Tyrode's solution over a 90 min period, and representative isochrones maps are illustrated in Figure 1A, Upper Panel. CV_L_ and CV_T_ were calculated and are reported in Figure 1B.

In hearts perfused with control Tyrode's solution, both CV_L_ and CV_T_ isotropically increased over time. We hypothesized that the gradual CV increase over time was a result of either dye washout or degradation. Therefore, in order to compare CV at *t* = 0 and 90 mins without the effect of the dye, we delayed Di-4-ANEPPS perfusion for 75 mins in another set of experiments. Importantly, Figure 1 demonstrates that CV in the delayed dye perfusion experiments was not statistically different from CV in the original and early dye perfusion experiments. Further, CV still increased in the delayed dye perfusion experiment over the additional 90 mins (Figure 1). The percent increase in CV_L_ and CV_T_ over the first and second 90 min durations was similar (Figure 1C). Taken together, these data suggest that the observed rise in CV is related to dye washout or degradation.

**Figure 2:**
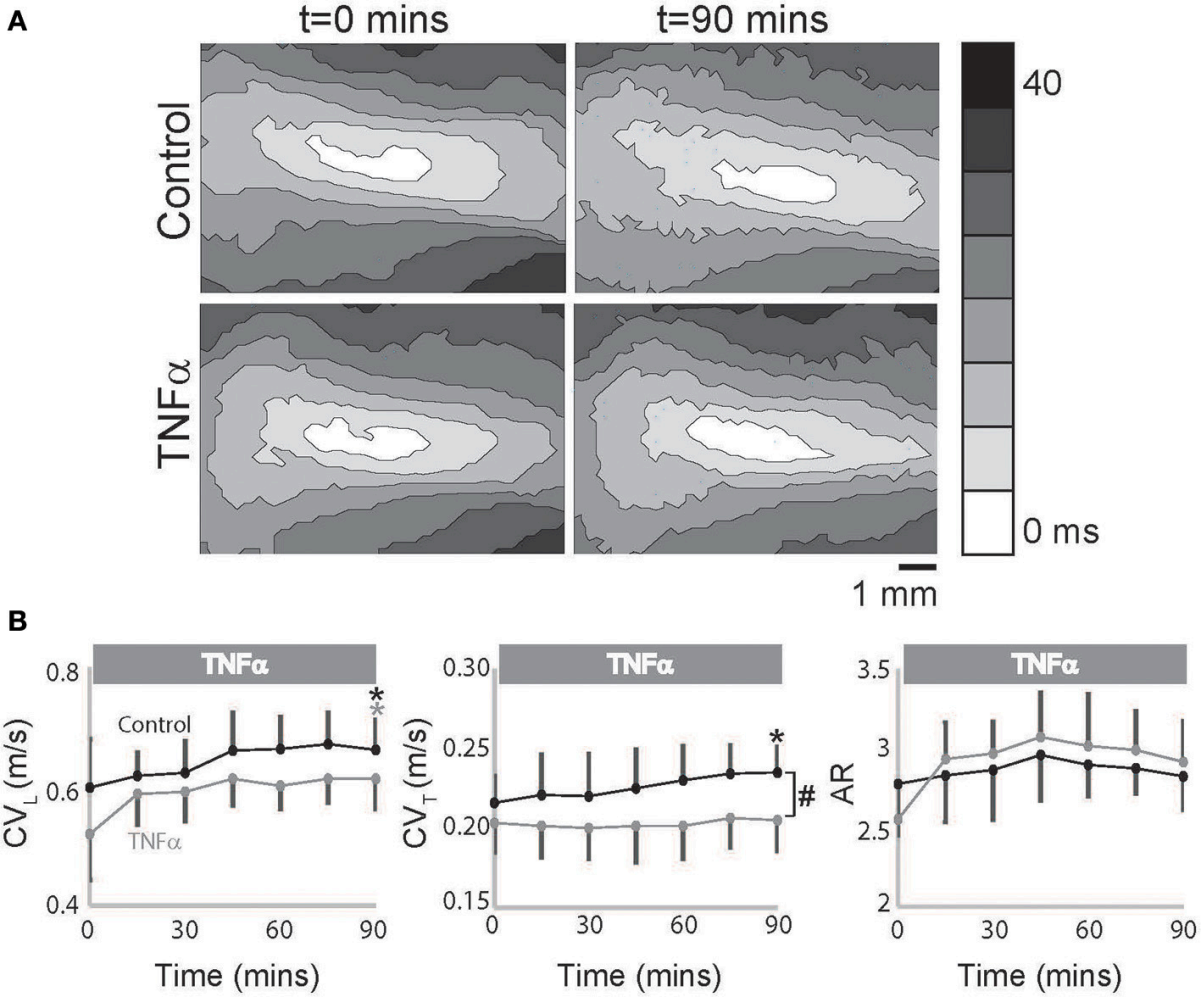
**TNFα slows conduction. (A)** Representative isochrones maps from control and TNFα perfused hearts at *t* = 0 and 90 mins. **(B)** Summary of CV_L_, CV_T_, and AR values calculated from the optical recordings are graphed. Black and gray ^*^Indicates *p* < 0.05 between *t* = 0 and 90 mins in control and TNFα perfused hearts respectively by paired comparison. ^#^Indicates *p* < 0.05 between control and TNFα (unpaired).

However, in TNFα perfused hearts, CV_L_ alone increased over time with no change in CV_T_ as seen in representative isochrones (Figure 2A) and summary data (Figure 2B), and as a result an increasing trend in AR was also observed (*p* = 0.055, Figure 2B). Additionally, CV_T_ was significantly slower in TNFα perfused hearts relative to control at *t* = 90 mins. Taking into consideration the temporal effects of dye on our measurements as demonstrated above, the data suggest that TNFα slows CV_T_ over 90 mins. We therefore conclude that the presence of TNFα in the perfusate slows CV, preferentially in the transverse direction, and increases conduction anisotropy.

### Conduction velocity-control vs. TNFα + high calcium

**Figure 3:**
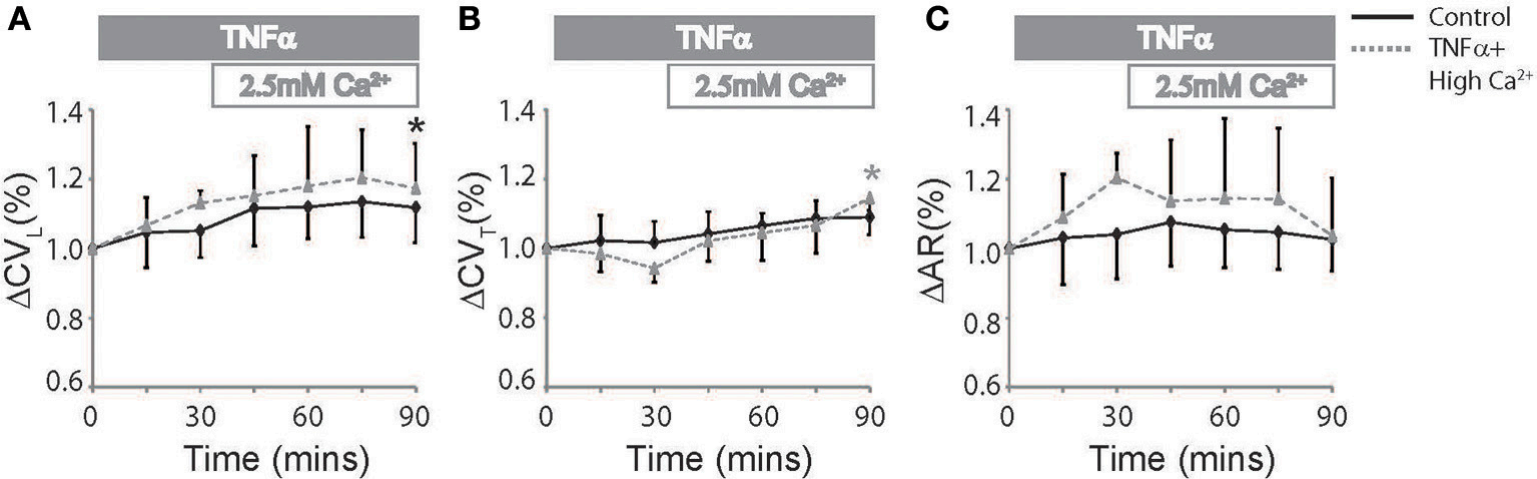
**Conduction rescue by high calcium**. Percent change in CV_L_ **(A)**, CV_T_ **(B)**, and AR **(C)** over time induced by control Tyrode perfusion and hearts treated with TNFα + high calcium at *t* > 30 mins. Black and gray ^*^Indicates *p* < 0.05 between *t* = 0 and 90 mins in control and TNFα perfused hearts respectively by paired comparison.

Elevating extracellular calcium has been demonstrated to acutely increase CV possibly by improving EpC (George et al., 2015, 2016). We next perfused TNFα treated hearts with a physiologically high calcium solution (2.5 mM) introduced 30 mins into the experiment to determine if elevating extracellular calcium can attenuate TNFα induced CV slowing. Percent changes in CV_L_, CV_T_, and AR are reported in Figure 3 to compare the effects of TNFα + high calcium to control Tyrode perfused hearts. At *t* = 90 mins, CV_L_ significantly increased relative to *t* = 0 mins and was similar to control at *t* = 90 mins. This suggests that neither TNFα nor high calcium have a significant impact on CV_L_, though the change in CV_L_ may be below our ability to detect. On the other hand, CV_T_, as illustrated in Figure 3B, began to separate from control after the initial 30 mins of just TNFα perfusion, suggesting that TNFα can acutely slow CV_T_. Interestingly, elevating calcium in the presence of TNFα acutely increased CV_T_ within 15 mins and restored CV_T_ back to control values at 90 mins. Additionally, CV_T_ was significantly greater at *t* = 90 mins relative to 0 mins during TNFα + high calcium. Finally, AR was not significantly different in control or TNFα + high calcium perfused hearts at *t* = 90 mins. Taken together, these data demonstrate that high calcium restored CV in TNFα treated hearts to control values.

### Action potential

**Figure 4:**
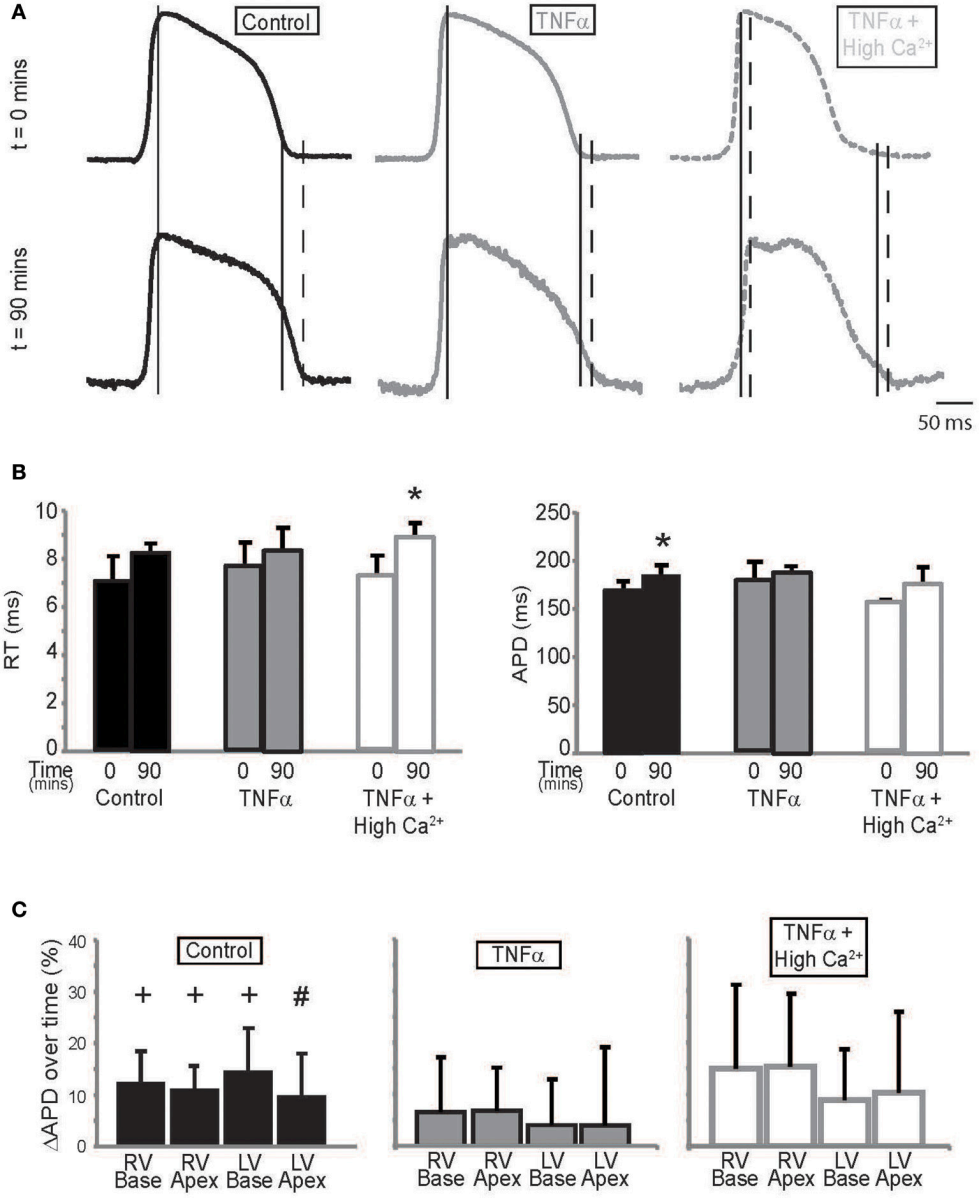
**Action potential parameters (A)** Representative action potentials recorded from the anterior epicardium of control, TNFα, and TNFα + high calcium perfused hearts. Solid long lines indicate peak of AP relative to the time aligned beginning of the action potential. Solid and dashed short lines indicate fiducials in upper and lower panels respectively (*t* = 0 and 90 mins) and **(B)** Summary of RT (left) and APD (right) calculated from these hearts at *t* = 0 and 90 mins. **(C)** APD quadrant analysis for hearts perfused with control Tyrode's, TNFα and TNFα + high calcium. ^*^Indicates *p* < 0.05 relative to *t* = 0 mins by paired comparison. + indicates *p* < 0.05 and ^#^Indicates *p* = 0.05 relative to zero.

TNFα can modulate several ionic currents in the heart (Grandy and Fiset, 2009; Guillouet et al., 2011). We therefore quantified action potential parameters such as rise time (RT) and APD to determine the effects our interventions had on cardiac electrophysiology. RT was not significantly different between *t* = 0 and 90 mins during control or TNFα perfusion. However, RT increased at *t* = 90 mins with TNFα + high calcium. This can be seen in Figure 4A, and summary data is presented in Figure 4B, left panel.

APD, on the other hand, is significantly prolonged over time during control Tyrode perfusion but this effect was not as pronounced in the presence of TNFα or TNFα + high calcium (Figure 4B, Right Panel) suggesting that TNFα may be modulating ionic currents that determine APD. The optically mapped region on the anterior epicardial surface was then divided into 4 quadrants corresponding to the right ventricular (RV) base and apex, and the LV base and apex. APD was compared to determine if the effects of TNFα and TNFα + high calcium were homogenous (Figure 4C) across the epicardial surface. Although, APD was significantly prolonged only in 3 of 4 quadrants in control Tyrode's perfused hearts, there were no significant APD changes in hearts perfused with TNFα or TNFα + high calcium hearts. Furthermore, no significant changes in APD were observed between quadrants with any intervention suggesting homogenous APD modulation.

### ECG

**Figure 5:**
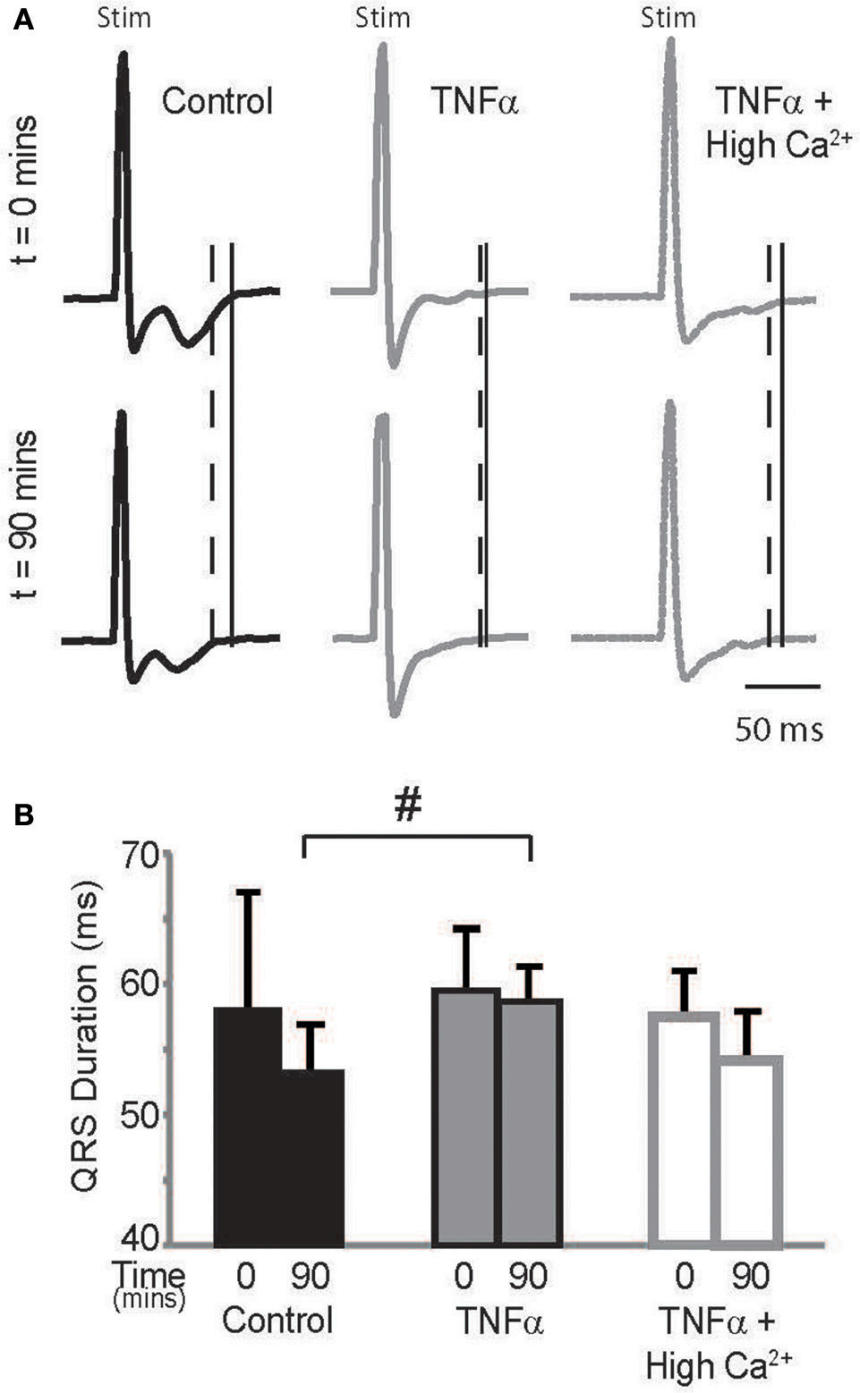
**ECG (A)** Paced QRS complexes from volume-conducted ECG traces recorded from control, TNFα and TNFα + high calcium perfused hearts. Solid vertical lines indicate end of QRS of ECGs from *t* = 0 mins and dashed vertical lines indicate end of QRS and T waves of ECGs from *t* = 90 mins. “Stim” indicates stimulus artifacts. **(B)** Summary of QRS duration. ^#^indicates *p* < 0.05 relative to control (unpaired).

Optical maps were obtained from the anterior epicardial surface of the heart from a region that was ~16 × 16 mm. The CV, RT, and APD parameters reported above are based on changes in this specific field of view. However, several conditions can result in a heterogeneous modulation of CV which then increases risk for arrhythmias (Gutstein et al., 2001; Poelzing and Rosenbaum, 2004). Therefore, the ECG was assessed during pacing to determine QRS duration, which would indicate if the effect of TNFα on CV was also observed at the whole heart level. Representative ECG traces recorded from control, TNFα, and TNFα + high calcium treated hearts are presented in Figure 5A, and QRS durations are reported in Figure 5B. Changes in QRS duration over time (*t* = 0 to 90 mins) were not significantly different with any of the interventions. However, QRS duration at *t* = 90 mins was significantly prolonged in TNFα perfused hearts relative to control, consistent with CV slowing observed with TNFα relative to control reported above. Also, QRS duration was similar between control and TNFα + high calcium treated hearts at *t* = 90 mins further suggesting that elevating calcium improves conduction in the presence of TNFα.

### Perinexus

**Figure 6:**
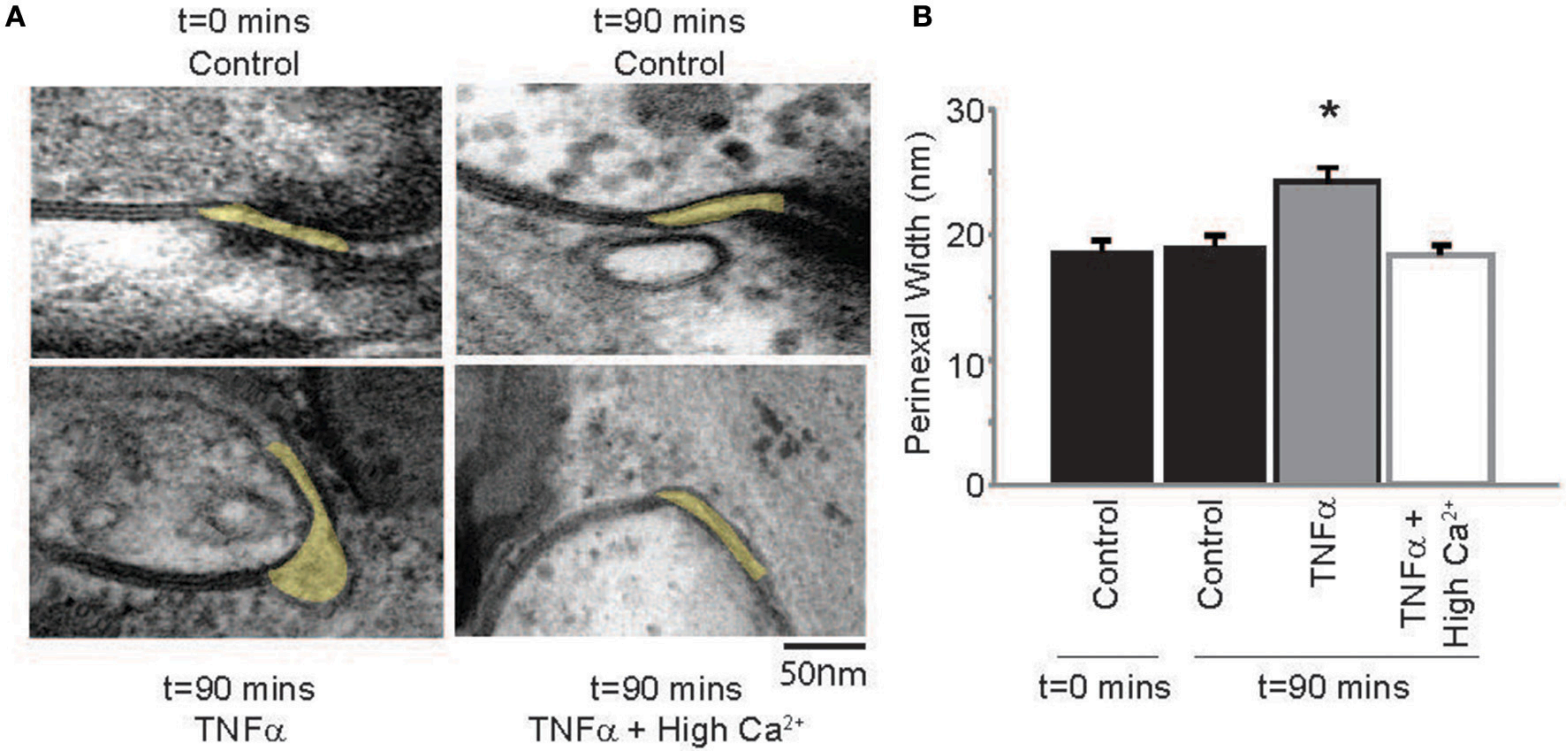
**TNFα modulates perinexal width (A)** Electron micrographs of representative perinexi (highlighted in yellow) from hearts treated with control, TNFα or TNFα + high calcium over 90 mins. **(B)** Average perinexal width. ^*^Indicates *p* < 0.05 relative to *t* = 0 (unpaired comparison).

Next, the effect of TNFα on proposed modulators of ephaptic coupling like the perinexus was determined, since we previously demonstrated that elevating calcium within the physiologic range can decrease perinexal width in mouse ventricular myocardium (George et al., 2015, 2016). Perinexal width (W_P_) was not significantly different at *t* = 0 or 90 mins in control Tyrode perfused hearts, but TNFα significantly increased W_P_ over the same time course (Figure 6). Elevating calcium in the presence of TNFα reduced W_P_ back to control values. This is consistent with our previous mouse study, where we demonstrated that increasing extracellular calcium decreases perinexal width. These data suggest that W_P_ correlates with observed CV changes induced by TNFα or TNFα + high calcium, and are consistent with predictions of EpC.

### Connexin43 expression, phosphorylation, and distribution

**Figure 7:**
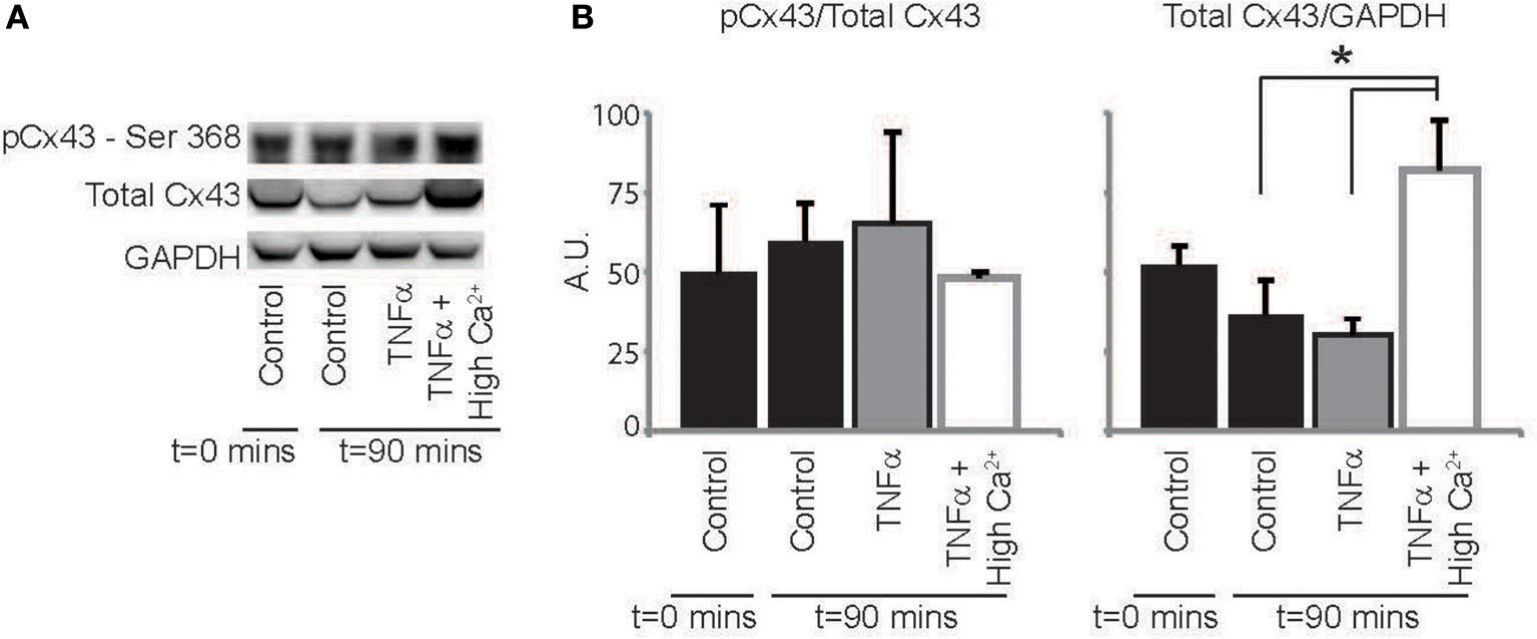
**Cx43 expression and phosphorylation modulation by TNFα (A)** Representative images of membranes blotted for total Cx43, Cx43 phosphorylated at Ser368 and GAPDH as a loading control. **(B)** Protein expression in hearts treated with control, TNFα and TNFα + high calcium at *t* = 0 and 90 mins. ^*^Indicates *p* < 0.05 by unpaired comparison.

Finally, we sought to determine if TNFα alters Cx43 protein expression, phosphorylation or distribution. Representative western blots in Figure 7A and summary data in Figure 7B demonstrate that total Cx43 and the ratio of pCx43/Cx43 was not significantly altered over 90 mins with either control Tyrode or TNFα perfusion. Interestingly, elevating extracellular calcium with TNFα significantly increased total Cx43 expression relative to TNFα alone, but the ratio of pCx43/Cx43 did not change. Therefore, though Cx43 modulation may not contribute to CV slowing by TNFα, improving GJ coupling may be a mechanism that contributes to CV preservation with high calcium at 90 mins.

**Figure 8:**
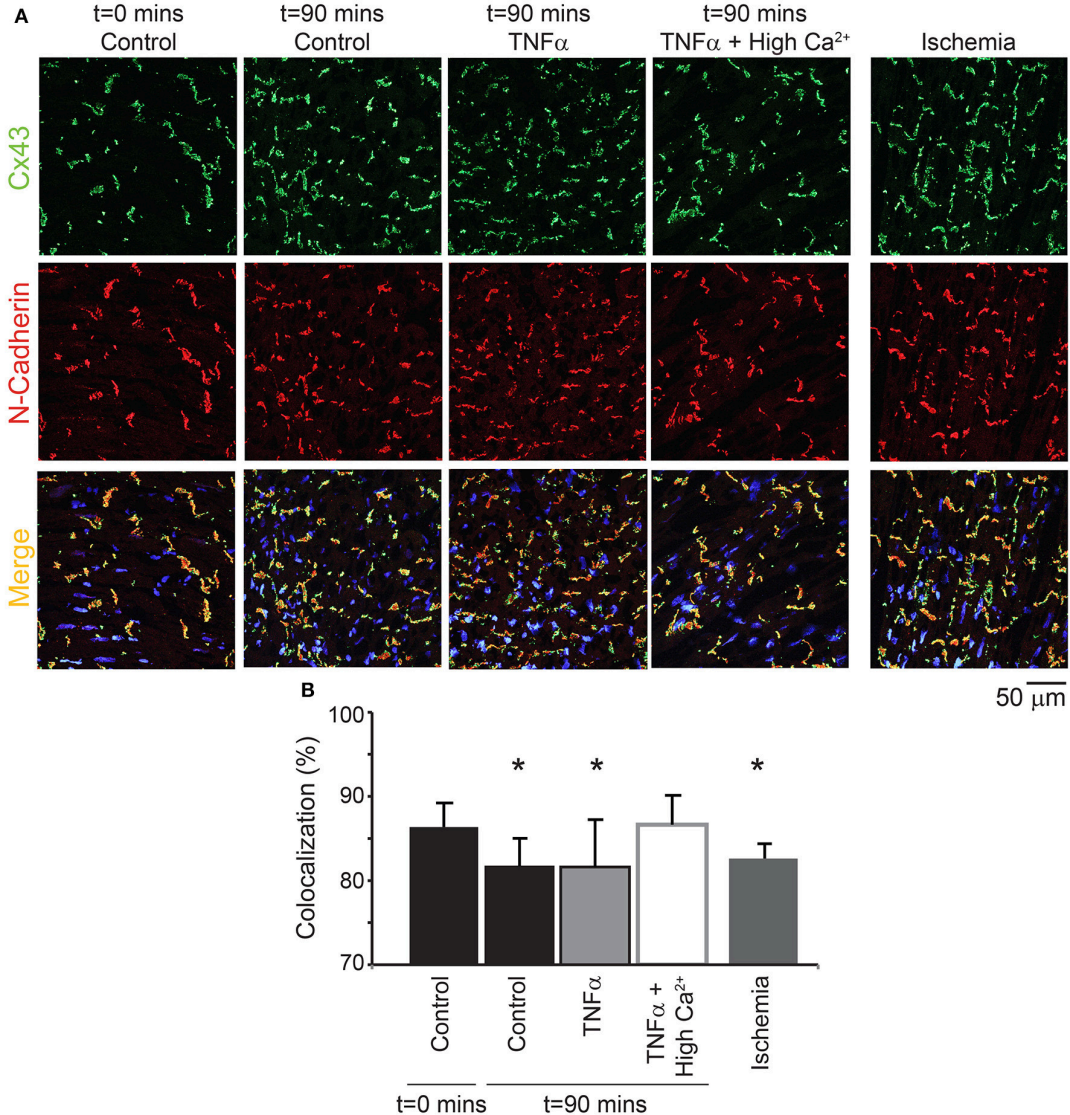
**Cx43 distribution modulation by TNFα (A)** Representative samples stained for Cx43 (green) and N-Cadherin (red, to mark the intercalated disc). The colocalization of the two signals is indicated in yellow. Samples from control, TNFα, and TNFα + high calcium were compared and 1 h of no flow ischemia was used as a positive control. **(B)** Summary data of percent Cx43 colocalized with N-Cadherin. ^*^Indicates *p* < 0.05 relative to *t* = 0 mins by unpaired comparison.

Next, the distribution of Cx43 was also quantified to determine if TNFα causes Cx43 remodeling over 90 mins (Figure 8). Although Cx43 expression was not significantly altered over time with control Tyrode or TNFα, co-localization of Cx43 with the intercalated disc protein N-Cadherin was significantly reduced for both interventions. This suggests that Cx43 redistributes around the myocyte over 90 mins in these isolated heart preparations. Finally, TNFα + high calcium did not alter total Cx43 expression as mentioned above, and Cx43 distribution around the myocyte at *t* = 90 mins was preserved similar to control at *t* = 0 mins.

Lastly, as a positive control, hearts were exposed to 1 h of no flow ischemia. In these hearts, Cx43 co-localization with N-Cadherin was significantly reduced relative to control suggesting redistribution from the intercalated disc, as expected from previous publications (Smith et al., 1991; Huang et al., 1999; Jain et al., 2003).

## Discussion

Conduction is a multifactorial phenomenon that can be modulated by several factors including tissue architecture, intercellular coupling and excitability of the tissue. Tissue architecture includes cellular dimensions and extracellular space, both extracellular volume and composition (fibrosis). Two modes of intercellular electrical coupling have currently been suggested in cardiac tissue, electrotonic coupling mediated by gap junctions and electric field coupling at ephapses. While modulating the gap junctional proteins, such as Cx43, can alter gap junctional coupling, altering the width of the perinexus, and/or its ionic composition has been suggested to alter ephaptic coupling. Finally, excitability of the tissue can be modulated by altering ion channel functional expression. In this study, we investigated the role of several of these determinants of cardiac conduction in modulating CV during acute TNFα exposure.

Hearts were exposed to 100 pg/ml TNFα, similar to concentrations previously reported in cardiac tissue during diseases like end-stage dilated cardiomyopathy and ischemic heart disease. (Giroir et al., 1992; Torre-Amione et al., 1996) We sought to determine whether acute TNFα exposure over 90 mins was associated with changes in CV modulators like ephaptic and/or GJC. Briefly, CV slowing was observed in TNFα perfused hearts relative to control and this was associated with perinexal expansion without a concomitant change in Cx43 expression or phosphorylation. Additionally, Cx43 cellular localization was altered by TNFα. Elevating extracellular calcium within guinea pig physiologic limits in the presence of TNFα rescued CV at 90 mins by restoring W_P_ to control values and improving GJ expression, phosphorylation, and distribution.

Another novel finding of this study was that after Di-4-ANEPPS perfusion and excess dye washout, CV increased over time up to 90 min. Addition of more Di-4-ANEPPS at 90 min reversed this increase and restored CV to initial values. However, a similar increase in CV over time was observed after an additional 90 min. This can be interpreted as acute CV slowing due to Di-4-ANEPPS perfusion and restoration of CV over time due to either washout or degradation of the dye. It was previously suggested that Di-4-ANEPPS slows CV due to inhibition the sodium-potassium ATPase (Fedosova et al., 1995; Larsen et al., 2012). Restoration of CV, as in the current study, may be an effect of reversal of the inhibition of the sodium-potassium ATPase. However, this requires further investigation.

### TNFα and ephaptic coupling

TNFα is one marker of acute inflammation and is important to many physiological processes. One important effect of TNFα is its ability to modulate vascular leakiness as evidenced by studies demonstrating that elevated TNFα concentrations increase vascular permeability (Hansen et al., 1994). Increased vascular permeability can then lead to extracellular edema formation in tissue (Logstrup et al., 2015). In the heart, extracellular edema has been demonstrated to slow CV and increase arrhythmogenesis possibly due to reduced ephaptic coupling between myocytes (George and Poelzing, 2015; George et al., 2015; Veeraraghavan et al., 2015; George et al., 2016).

In addition to gross extracellular edema, the results of this study indicate that TNFα can increase extracellular volumes in restricted nanodomains within intercalated discs, like the perinexus. Fluid retention in the bulk extracellular space could be one causative factor of TNFα-induced perinexal edema. Another explanation for perinexal expansion could be the effect of TNFα on structural junction proteins along the intercalated disc. For example, TNFα can reduce N-Cadherin (Celes et al., 2007) and plakoglobin (Asimaki et al., 2011) expression in the heart, which are essential components of the structural junctions that hold the two adjacent membranes together. Structurally uncoupling these junctions could also increase intercellular separation at the perinexus and thereby cause perinexal edema.

Finally, structural proteins like N-Cadherin, desmoglein, and desmocollin have calcium sensitive domains that determine binding affinity (Chitaev and Troyanovsky, 1997; Vleminckx and Kemler, 1999). Hypocalcemia and calcium-free solutions have been demonstrated to cause intercalated disc dehiscence by reduced binding affinity of these proteins (Chitaev and Troyanovsky, 1997; Vleminckx and Kemler, 1999). This can result in perinexal widening. Additionally, we recently demonstrated that increasing extracellular calcium can reduce perinexal width and maintain the structural integrity of the ephapse possibly by similarly modulating the structural protein binding affinity (George et al., 2016). In this study, elevating extracellular calcium could enhance adhesion at these junctions during TNFα exposure, thereby restoring W_P_ to control values.

### TNFα and gap junctional coupling

While the effects of TNFα on Cx43 expression have been extensively studied, some groups report that TNFα reduces (Celes et al., 2007; Sawaya et al., 2007) or increases (Liu et al., 2012) Cx43 expression. Some factors that can explain these diverse results are the type of tissue studied, concentration of TNFα, period of exposure or other yet to be explored experimental differences (Celes et al., 2007; Sawaya et al., 2007; Kimura and Nishida, 2010; Liu et al., 2012). Additionally, TNFα has also been demonstrated to reduce Cx43 phosphorylation at serine 368 in anterior pituitary cells (Meilleur et al., 2007), which is important in modulating Cx43 GJ channel conductance. Cx43 remodeling and lateralization has also been reported in the atria of TNFα overexpressing mice (Sawaya et al., 2007).

In this study, we explored the effect of TNFα on Cx43 expression at a time scale (90 mins) significantly shorter than previous studies. Total Cx43 expression and the ratio of phosphorylated to total Cx43 was not significantly different in the presence of TNFα relative to control at 90 mins. Interestingly, the distribution of Cx43 around the myocyte was heterogeneously altered even within anterior left ventricular (LV) tissue samples analyzed here. This finding is similar to that observed in the atria of TNFα overexpressing mice where Cx43 expression was not altered but Cx43 was redistributed around the myocyte (Sawaya et al., 2007).

Interestingly, elevating extracellular calcium to 2.5 mM in the presence of TNFα improved GJ coupling. Previous studies have demonstrated that increasing intracellular calcium can decrease GJ coupling between cells (Maurer and Weingart, 1987; Kurebayashi et al., 2008) by dephosphorylating Cx43 by a Ca^2+^/Calmodulin pathway (Xu Q. et al., 2012). However, calcium concentrations used in other previous studies were significantly greater than the concentration used in this study. Our previous study in mice demonstrated that modulating calcium in the range used here did not affect Cx43 expression, phosphorylation or function over 30 mins (George et al., 2016). However, here we report that in the presence of TNFα, GJ coupling in guinea pig hearts improved when calcium was increased to 2.5 mM for 60 mins.

Calcium and TNFα have been identified as key cell signaling regulators of protein transcription including Cx43. For example, elevated calcium can activate MAPK–dependent pathways that have been reported to either increase (Squecco et al., 2006; Stanbouly et al., 2008) or decrease (Petrich et al., 2002) Cx43 expression and phosphorylation. In this study, the specific mechanisms underlying enhanced GJ coupling in guinea pig hearts in the presence of TNFα + high calcium is not fully understood and may involve the activation of one or more of these cell signaling pathways.

### TNFα and ionic currents

Ventricular heterogeneities of membrane proteins that form ion channels in the myocardium is well-established (Di Diego et al., 1996; Veeraraghavan and Poelzing, 2008), and TNFα modulates a variety of these ionic currents (Grandy and Fiset, 2009; Guillouet et al., 2011). In this study, APD prolongation was observed over 90 mins in control hearts but not in the presence TNFα with or without high calcium. APD prolongation in the control hearts could be a result of the phototoxic effects of Di-4-ANEPPS over time. (Schaffer et al., 1994; Hardy et al., 2009) In the presence of TNFα, several studies have reported that repolarizing potassium currents are reduced (Grandy and Fiset, 2009) which should theoretically increase APD. However, other studies have also demonstrated that inflammation is associated with APD shortening due to reduced L-type calcium current (Zhong et al., 1997; Greensmith and Nirmalan, 2013). In this study, the lack of APD prolongation with TNFα could be due to similar modulation of ionic currents.

Lastly, elevating calcium was also associated with increased action potential RT, suggesting that calcium decreased membrane excitability despite increasing conduction velocity. Increased RT could have been the effect of (1) decreasing sodium currents which would manifest as changes in the maximum rate of rise of the action potential (dV/dtmax) or (2) modulating diastolic membrane potential during the initial phase of excitation which can indirectly affect sodium channel availability. Calcium can modulate sodium channel phosphorylation and gating by a calcium/calmodulin kinase II dependent pathway (Wagner et al., 2006) and a five-fold increase in intracellular calcium has been reported to increase late sodium current (Wagner et al., 2006). However, the effects of physiologically elevating extracellular calcium, as we did in this study, on fast sodium current during cellular depolarization are unknown. The complex result that elevated calcium can increase RT while increasing CV requires further investigation. Finally, it is also possible that the increased GJ coupling in these hearts, with TNFα + high calcium, provides a greater sink to the excitatory current, thereby increasing RT. However, this theory also requires additional investigation.

## Limitations

Most of the analysis described above involves only the anterior epicardial region of guinea pig hearts and TNFα could be having different effects on cellular functioning in different regions of the heart. Transmural and interventricular differences in several parameters like protein expression and APD have been previously described (Grandy and Fiset, 2009; Guillouet et al., 2011) and amplification of these differences by factors like TNFα needs to be better understood in order to identify therapeutic options to treat cardiac conduction slowing caused by inflammation. Nonetheless, this study is the first to highlight that acute TNFα exposure detrimentally affects CV in ventricles and identifies perinexal and gap junction remodeling as potential underlying mechanisms.

Finally, TNFα is one of the many cytokines that are involved in the inflammatory process. Several others like IL-6, IFNγ, and IL-8 have all been identified as physiologic modulators during inflammation. In this study, we focused on understanding the effects of individually modulating only TNFα. This is an important step prior to identifying the cumulative effects of inflammatory factors that occur during the complex process of myocardial inflammation. Furthermore, TNFα inhibition has also developed as a therapy for diseases associated with inflammation (Moe et al., 2004; Buyukakilli et al., 2012), which also increases the significance of understanding how TNFα and its inhibitors may affect cardiac functioning.

## Conclusions

TNFα upregulation during inflammation can have significant effects on cardiac electrophysiology which includes anisotropic conduction slowing. Altering the perfusate calcium composition has been identified as a means to conceal the effects of TNFα on cardiac conduction acutely. Importantly, increasing extracellular calcium concentration in guinea pig hearts improves both proposed forms of electrical coupling between cardiac myocytes—Ephaptic and GJC and preserves cardiac conduction during acute TNFα exposure.

## Author contributions

SG: experimental design, data acquisition, analysis and interpretation, drafting manuscript, and approval. PC: data acquisition, analysis and interpretation, manuscript editing, and approval. RG: data interpretation, manuscript editing, and approval. JS: Data interpretation, manuscript editing, and approval. SP: experimental design, data interpretration, manuscript editing, and approval.

## Funding

This work was supported by an R01-HL102298 awarded to SP and R01-HL056728 awarded to RG, a VTCRI Medical Research Scholar Award, an American Heart Association Pre-doctoral fellowship, and the David W. Francis and Lillian Francis Scholarship Fund awarded to SG.

### Conflict of interest statement

The authors declare that the research was conducted in the absence of any commercial or financial relationships that could be construed as a potential conflict of interest.
